# Evaluation of Contrast Extravasation as a Diagnostic Criterion in the Evaluation of Arthroscopically Proven HAGL/pHAGL Lesions

**DOI:** 10.1155/2014/283575

**Published:** 2014-11-03

**Authors:** Catherine Maldjian, Vineet Khanna, James Bradley, Richard Adam

**Affiliations:** ^1^Department of Radiology, University of Pittsburgh, Pittsburgh, PA 15213-2582, USA; ^2^Department of Orthopedic Surgery, UPMC, Pittsburgh, PA 15213-2582, USA

## Abstract

*Purpose*. The validity of preoperative MRI in diagnosing HAGL lesions is debated. Various investigations have produced mixed results with regard to the utility of MRI. The purpose of this investigation is to apply a novel method of diagnosing HAGL/pHAGL lesions by looking at contrast extravasation and to evaluate the reliability of such extravasation of contrast into an extra-articular space as a sign of HAGL/pHAGL lesion. *Methods*. We utilized specific criteria to define contrast extravasation. We evaluated these criteria in 12 patients with arthroscopically proven HAGL/pHAGL lesion. We also evaluated these criteria in a control group. *Results*. Contrast extravasation occurred in over 83% of arthroscopically positive cases. Contrast extravasation as a diagnostic criterion in the evaluation of HAGL/pHAGL lesions demonstrated a high interobserver degree of agreement. *Conclusions*. In conclusion, extra-articular contrast extravasation may serve as a valid and reliable sign of HAGL and pHAGL lesions, provided stringent criteria are maintained to assure that the contrast lies in an extra-articular location. In cases where extravasation is not present, the “J” sign, though nonspecific, may be the only evidence of subtle HAGL and pHAGL lesions. *Level of Evidence*. Level IV, Retrospective Case-Control series.

## 1. Introduction

Current knowledge is mixed with regard to the reliability of MRI for diagnosing HAGL/pHAGL lesions. Specific findings on MRI that have been discussed previously include direct identification of the disrupted ligament, contrast extravasation medial to the humerus, and the “J” sign. This investigation looks at areas of contrast extravasation into anatomical spaces not previously studied, such as the quadrilateral space and intra/paramuscular spaces. This greatly enhanced the diagnostic capability of MRI. While contrast extravasation medial to the humerus has previously been elucidated, this is the first study to prescribe rigorous MRI criteria in order to differentiate true extravasation of contrast from a low-lying axillary pouch or “J” sign. While the “J” sign is not specific for HAGL/pHAGL, it was the only finding in 2 patients in our HAGL/pHAGL cohort, and it may be the only finding in subtle cases. This constitutes the largest series of arthroscopically proven HAGL lesions reported thus far. This is also the only series that evaluates interobserver variability for diagnosing HAGL/pHAGL by MRI criteria and the only study that evaluates a cohort of control patients for comparison to validate the use of an MRI sign of HAGL/pHAGL lesion. Our hypothesis is that extra-articular contrast extravasation will serve as a reliable diagnostic marker in the detection of HAGL/pHAGL lesions.

## 2. Methods

This study was performed retrospectively with internal institutional review board approval. Informed consent by the IRB was waived since this was a retrospective study. A list of exams performed during a seven-year period was extracted from the institutional database conforming to the following criteria: arthroscopically proven HAGL lesion with preoperative MRI. 12 cases were retrieved that satisfied these criteria. 11 of these studies were performed as MR arthrograms. One was performed as a nonarthrographic, conventional MRI. A control cohort of 23 patients was retrieved from the institutional database over a one-year period that had MR arthrogram and in addition had available arthroscopic data confirming that there was no evidence of HAGL or pHAGL. Arthrographic technique was performed with an anterior approach, instilling 12 mL of highly diluted gadolinium based contrast (0.1 mL/20 mL or 5 parts per 10,000). The mixture contained 10 mL of saline, 5 cc of iodinated contrast (Isovue 300, (iopamidol)), 5 cc of 1% lidocaine, and 0.1 mL of gadobenate dimeglumine (Multihance, Bracco Diagnostics, Princeton, NJ). MRI exams were all performed on 1.5 Tesla units. Examinations were performed supine using a dedicated shoulder coil. Standard oblique sagittal T1 and fat saturated axial and oblique coronal T1 weighted pulse sequences were obtained. Fat suppressed T2 weighted acquisitions in all 3 imaging planes were also obtained in all arthrographic cases. In the one nonarthrographic case, axial PD, oblique coronal PD and fat suppressed T2, and oblique sagittal T1 and fat suppressed T2 imaging was performed. The images were evaluated retrospectively, independently by 2 fellowship trained musculoskeletal radiologists, each with over 10 years of experience, for extravasation of contrast into an extra-articular space. Criteria for extravasation would normally include any space that does not communicate with the joint; however, we excluded bursal spaces in our analysis, such as the subdeltoid/subacromial bursa and subcoracoid bursa, which would have other clinical ramifications, and we ensured that none of the cases demonstrated overdistension of the joint where anterior leakage could obfuscate the diagnosis of true pathological extravasation. Criteria for extravasation included visualization of contrast in intramuscular spaces, inter/paramuscular spaces, the quadrilateral space, and the juxta-diaphyseal region along the humeral shaft. Extravasation into the latter space was defined more rigorously than on prior studies and consisted of contrast extending along the medial humeral shaft beyond the field of view, such that no pouch like structure maintaining the contrast was present in the field of view. This was done intentionally in order to explicitly differentiate such extravasation from the “J” sign as well as from possible overdistension, both of which preserve an axillary pouch structure. In the one patient with a nonarthrographic exam, joint fluid extending off the field of view constituted criteria for juxtahumeral leakage. Fluid in the quadrilateral space was designated as positive criteria for “extravasation,” since this is not normally seen after trauma. Intramuscular or paramuscular edema, on the other hand, can be seen after trauma; therefore “extravasation” for this parameter in the nonarthrographic case was defined as intra- or paramuscular fluid confluent with and communicating with joint fluid. The presence or absence of extravasation was recorded for each reader and compared for agreement. The location of extravasation(s) was also specified independently by each reader.

After independent analysis of all 12 cases by 2 fellowship trained musculoskeletal radiologists, any disagreement between the 2 readers was resolved by consensus between both readers as a final analysis. We also evaluated for the presence of a “J” sign, which we defined as a low-lying axillary pouch. Since there is no method to quantify this in the current literature, we adhered to a qualitative analysis. Devising a method to quantify a low-lying axillary pouch with which we define the “J” sign would be beyond the scope of this paper, which seeks to address the usefulness of contrast extravasation for diagnosing IGL ruptures. Furthermore, this sign is very nonspecific as there are several etiologies for ligamentous laxity and stretching other than IGL tear, including injury without frank tear, and theoretically connective tissue disorders such as Marfan syndrome and Ehlers-Danlos syndrome, and idiopathic or genetic hypermobility. No correlative gold standard on arthroscopy exists with which we gauge the accuracy of a hypothetical quantitative measurement made on MRI to differentiate between these possibilities.

## 3. Results

**Figure 1:**
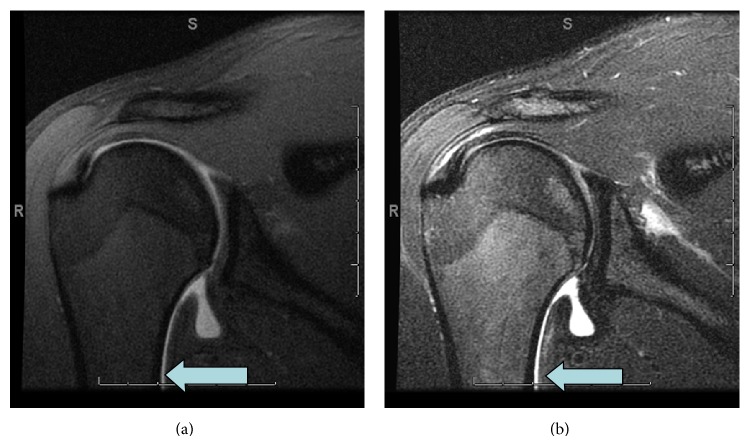
Case 8. Oblique coronal T1 and T2 demonstrating contrast/fluid extending along the medial humerus and off the field of view (arrow), consistent with extra-articular extravasation.

The age range for the patient population with HAGL/pHAGL injuries was 15–44. The mean age was 21.5. There were 6 males and 6 females. 8 of the lesions were right-sided and 4 were left-sided. At arthroscopy 7 HAGL lesions and 4 pHAGL were identified and one patient's shoulder exhibited both HAGL and pHAGL. 10 patients demonstrated extra-articular extravasation in at least one space according to both readers and 2 patients did not, with 100% agreement between both readers as to general extravasation in at least one space (Tables 1 and 2). Therefore the diagnosis of ligament rupture based on extravasation was achieved with excellent interobserver reliability with Kappa = 1. Further analysis of the discrete locations of extravasation demonstrated complete agreement for all except for one instance of quadrilateral space extravasation, with interobserver reliability by kappa statistics of 1 for juxtahumeral extravasation, kappa statistic of 1 for intramuscular extravasation, kappa statistic of 1 for paramuscular extravasation, and kappa statistic of 0.883 for the quadrilateral space. The one case of discordant readings involved extravasation into the quadrilateral space in the one nonarthrographic case (Table 2). The correct diagnosis of ligament rupture was achieved by both readers in this case due to the concomitant presence of juxta-diaphyseal leakage. Reanalysis of this case by consensus resulted in a final agreement of extravasation of fluid into the QL space. Juxta-diaphyseal contrast was seen in 2 cases and juxta-diaphyseal fluid communicating with the joint was seen in the one nonarthrographic case where it was deemed the equivalent of extravasation, yielding a total of 3 cases of juxta-diaphyseal leakage (Figure 1).

**Figure 2:**
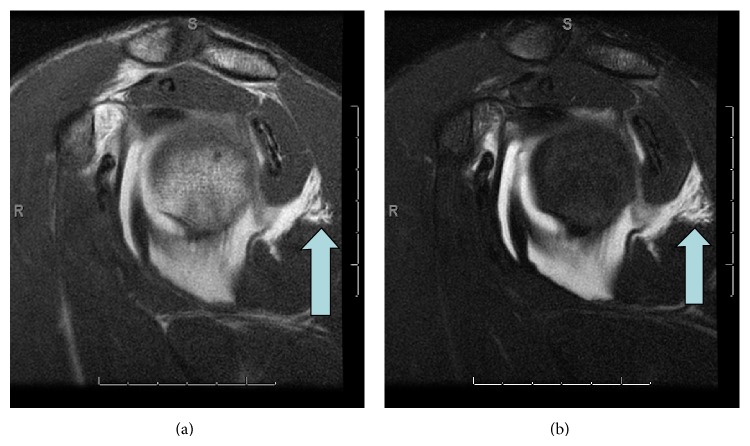
Case 6. Oblique sagittal T1 and T2 demonstrate contrast/fluid extravasating between muscle fibers of teres minor (arrow).

**Figure 3:**
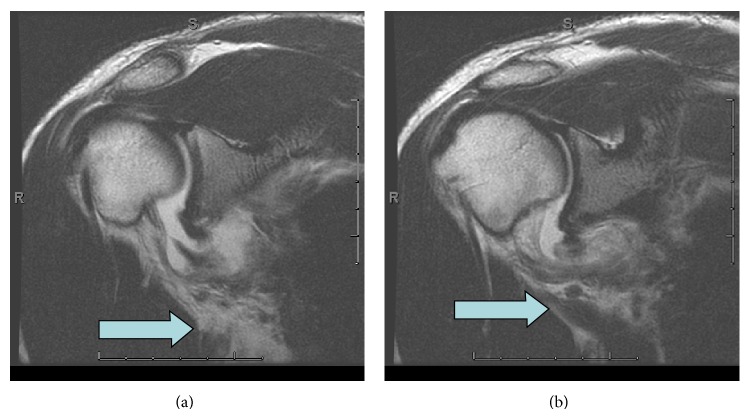
Case 11. Oblique coronal T2 demonstrates joint fluid extending into quadrilateral space (arrow).

**Figure 4:**
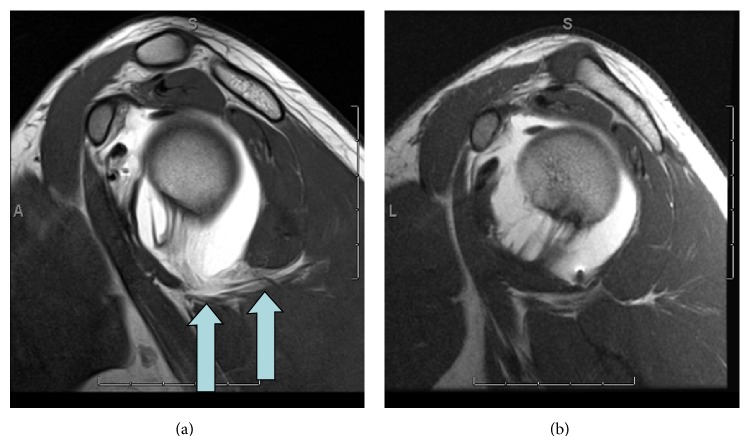
Case 9. Oblique sagittal T1 before and after HAGL repair: (a) prerepair shows contrast/fluid in quadrilateral space. (b) shows no extra-articular extravasation 2 years after repair. The intermuscular distance between the teres major and teres minor is increased on (a) (arrows) with an apparent gap at the axillary pouch in this area, which is no longer seen after repair.

**Figure 5:**
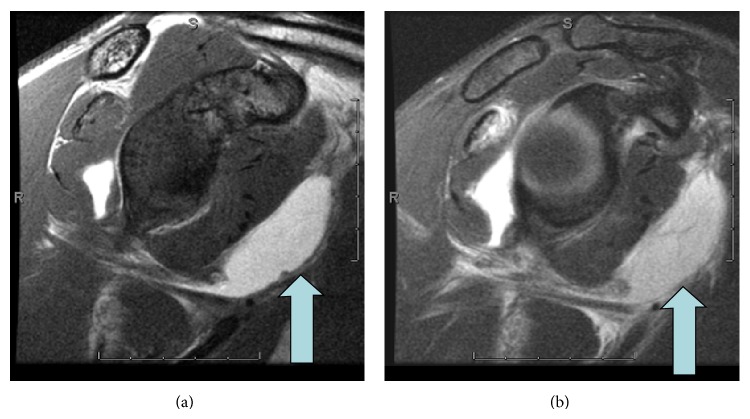
Case 10. Oblique sagittal T1 and T2 demonstrate contrast/fluid in the quadrilateral space and extending anterior to the subscapularis muscle (arrow).

Paramuscular/intramuscular extravasation was seen in 3 patients (Figure 2). All three patients had pHAGL. 2 demonstrated teres minor muscle extravasation, one with and one without intermuscular involvement between the teres minor and infraspinatus. The 3rd patient demonstrated leakage anterior to the subscapularis, remote from the typical location of extravasation through the anterior capsule from overdistension of the joint. Quadrilateral space extravasation was identified in 7 instances with one of these being the nonarthrographic study where the presence of fluid in the quadrilateral space was deemed the equivalent of contrast by one reader and subsequently by consensus by both readers (Figure 3). One of the 4 patients with pHAGL exhibited this finding. 5 of the 7 patients with HAGL exhibited this finding. The sole patient with both pHAGL and HAGL also exhibited this finding. Of the three locations (juxta-diaphyseal, para/intermuscular, and quadrilateral space) there was more than one location involved in any given patient in 3 patients of the 10 that showed extravasation, or 30%, and in 3 of the total patients with HAGL/pHAGL or 25%. If the spaces are further subdivided into juxta-diaphyseal, paramuscular, intermuscular, and quadrilateral space, more than one location of extravasation were seen in 4 of 10 patients with extravasation (40%) and in 4 of the total cohort of patients with HAGL/pHAGL or 33%. The most common site of extravasation overall was in the quadrilateral space, where it occurred in 7 of 10 patients who demonstrated extravasation or 70% and in 7 of the 12 patients with HAGL and/or pHAGL lesions or 58% (Figure 4). 3 of these also showed additional site of leakage, one in an inter/paramuscular space (anterior to the subscapularis) (Figure 5) and two in a juxta-diaphyseal location. Juxta-diaphyseal leakage occurred in 3 patients, two being in conjunction with quadrilateral space involvement and one occurring as an isolated finding. In all three patients, arthroscopy identified HAGL lesions. None of the patients with pHAGL exhibited this finding. There were 3 cases of para- or intramuscular involvement. As stated above, one of these 3 cases occurred in conjunction with quadrilateral space leakage. The other 2 cases were isolated findings, both demonstrating teres minor muscle infiltration.

**Figure 6:**
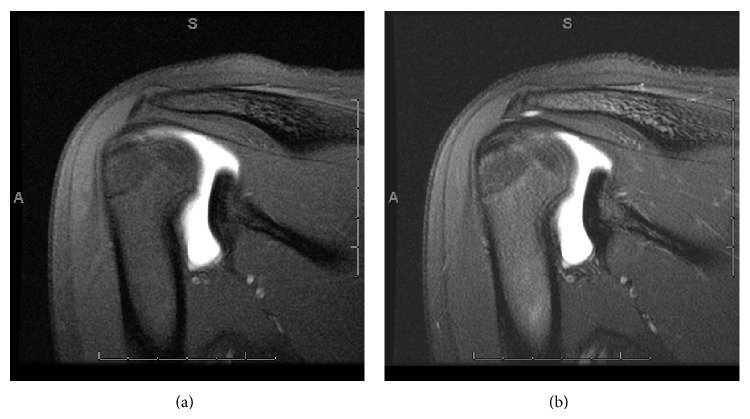
Case 12. HAGL not identified on initial report. Oblique coronal T1 fat saturated and oblique coronal T2 fat saturated images demonstrate J sign of MR arthrogram. No other findings were seen.

Correlation with the initial MRI reports at the time of the exams revealed that the diagnosis of HAGL/pHAGL was originally missed in 4 of the 12 cases. On retrospective review all 4 cases demonstrated extravasation and/or “J” sign. 2 of these had isolated “J” sign (Figure 6). 2 of these had a “J” sign in conjunction with quadrilateral space involvement.

**Figure 7:**
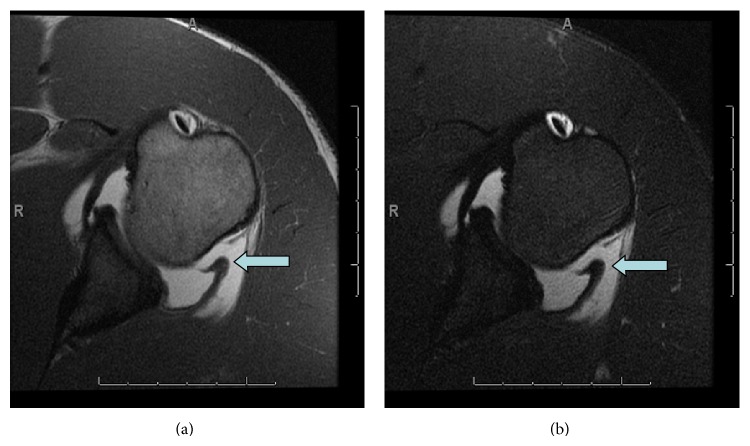
Case 7. Axial T1 and fat saturated T2 images demonstrate posterior band of IGL with thickening and retraction of ligament (arrow) and contrast on both intra- and extra-articular sides of the ligament.

Overall detection based on leakage of contrast/fluid was 10 out of the 12 patients or 83%. Direct visualization of a ruptured ligament was seen in 5 of the 12 patients with HAGL or pHAGL or 42% (Figure 7) and all of these cases demonstrated some form of extra-articular contrast extravasation. Therefore detection based on extra-articular contrast extravasation proved to be a superior method in comparison to the direct sign of ligament tear.

## 4. Control Cohort

The age range of the control population was 17 to 79. The mean age of the control population was 37.5. There were 10 females and 13 males. There were 12 right and 11 left shoulders evaluated. Results of MR arthrography analysis for both readers demonstrated no extravasation into the spaces designated for this study in the control population patients. There was 100% agreement between readers (Table 3).

## 5. Discussion

The IGL is the main anterior stabilizer of the shoulder at 90 degrees of abduction and external rotation and has an important role for glenohumeral joint stability [2]. Humeral avulsion of the inferior glenohumeral ligament was originally described by Nicola in 1942 [3]. HAGL contribute to anterior instability in less than 10% of cases [4, 5]. Nonetheless, they are important to recognize as they are accompanied by additional arthroscopic abnormalities in most cases [6]. Knowledge of a potential HAGL lesion prior to arthroscopy may be useful for preoperative planning and treatment [6]. If these lesions are not recognized and addressed when coexisting with other causes of instability, treatment of the other causes alone may result in unremitting instability [6]. Several investigators have expounded on imaging findings of HAGL lesions. Bone avulsion from the medial aspect of the humeral neck has been described as a radiographic finding [7]. This is reported to occur in 20% of cases [8]. Therefore, radiography is not sufficiently sensitive for diagnosing these lesions. Findings on MRI include extravasation of contrast through the defect, extravasation along the medial humeral shaft, [8, 9] and the J sign. The “J” sign refers to the appearance of the axillary pouch. When avulsion occurs in HAGL/pHAGL the detached end of the IGL falls inferiorly and converts the axillary pouch from its usual “U” shape to a “J” shaped morphology [8, 9].

The reliability of MRI to establish the diagnosis of HAGL varies widely from study to study. This apparent wide divergence in the reported reliability of MRI for diagnosing HAGL lesions was the impetus for our study.

The largest MRI study to date describes posterior HAGL lesions in 17 pts, 8 of which had arthroscopic confirmation [10]. Criteria for positive cases on MRI were detachment of the ligament with or without abnormal distribution of contrast along the humeral shaft based on coronal and sagittal images. All 8 lesions that had arthroscopy were confirmed [10]. Their study did not evaluate patterns of contrast extravasation in the 8 arthroscopically proven cases. In addition, the MR images were read by 2 radiologists in consensus, so that interobserver reliability is not established. No control cohort was utilized in their study to test for validity. Bui-Mansfield et al. reported on 4 patients with arthroscopy and MRI data, with 2 of the 4 positive cases being missed on MRI [8]. In addition, false positive MRIs were reported by these investigators. The MRI exams were not reported as MR arthrograms; therefore it is assumed that they were performed without intra-articular contrast. Castagna et al. reported on 9 de novo arthroscopically proven posterior HAGL lesions, with inclusion criteria consisting of no history of prior shoulder surgery [11]. Despite 77% of their patients undergoing MRI (7/9), pHAGL lesion was not detected on any of the preoperative MRI exams. All 7 of these MRIs were performed as MR arthrograms. None of the MRIs were diagnosed as pHAGL preoperatively. Interestingly, the authors reported that on retrospective review 6 of the 7 MRIs demonstrated findings consistent with pHAGL. No MRI criteria were stated for establishing the diagnosis of pHAGL lesions for the initial interpretation or for the retrospective review, but once the diagnosis was known arthroscopically, the findings became apparent in retrospective review. Therefore, it would seem that the diagnosis is attainable; however the criteria for achieving the proper diagnosis need to be better elucidated.

Melvin et al. reported on 4 cases which were deemed HAGL lesions on MRI but arthroscopically did not have HAGL [12]. 3 of the 4 MRI were performed as MR arthrograms and these 3 had capsular rents on arthroscopy. These authors state that the diagnosis of HAGL lesions should be reserved for arthroscopy. While certain familiar MRI criteria were utilized in that study, the application of these criteria was somewhat lax. In one illustration the authors use contrast alongside the medial humeral neck as a false positive diagnosis for HAGL. In that case the contrast appears to be suspended by an irregular pouch and is not free flowing down the humeral diaphysis and off the field of view. The investigators found a hole in the IGL on arthroscopy but no HAGL lesion with those imaging findings. We applied more stringent criteria for our definition of extra-articular extravasation of contrast along the humeral shaft in which we required the contrast to be free flowing off the field of view with no discernible pouch like structure maintaining the contrast, thereby assuring that the ligament was torn. In 2 additional cases those investigators state that the MRI demonstrates a tear of the IGL where both these images demonstrate low signal strands of tissue in the axillary pouch. One of these 2 cases had a posterior capsular rent without a HAGL lesion. One of the cases depicted what appeared to be a J sign. The finding of “disruption” of the ligament was unreliable in that study for diagnosing HAGL. Part of the problem is that the criteria for ligamentous disruption are not clearly defined and can be subjective. Relying solely on the appearance of what one believes is the ligament is problematic because non-HAGL capsular injuries may be difficult to differentiate from ligamentous tears on MRI.

In our study, we amassed arthroscopically proven cases of HAGL lesions, the largest such series to date. We applied specific criteria to these cases, placing our emphasis on extravasation of contrast into extra-articular spaces as a novel method to ensure that the ligament is truly disrupted. This is the first study to test interobserver reliability of an MR sign of HAGL/pHAGL injury.

When we embarked upon this study, we sought signs that would be more straightforward than the current methods for diagnosing IGL injuries. Contrast extravasation would be one such clearly definable, easily discernible, objective, and reproducible method of doing so. It is therefore not surprising that our study showed excellent interobserver variability, establishing this as a reliable method. The fact that no extravasation was seen in any of the control patients establishes validity of using contrast extravasation as a sign of HAGL or pHAGL injury and justifies this novel approach. No prior investigation has tested the reliability or validity of any MRI sign of HAGL/pHAGL lesions. Extravasation of contrast into anatomical spaces such as the quadrilateral and intra/paramuscular spaces has not been previously investigated for HAGL/pHAGL lesions. Furthermore, classical findings in anatomical spaces that have been previously described were classified more rigorously than on prior studies. Extension along the humeral diaphysis was deemed extra-articular extravasation only if it extended inferiorly beyond the field of view such that no pouch like structure could be seen restricting the free flow of contrast. Persistence of a low-lying pouch like structure was designated as a “J” sign and was classified as a separate and distinct finding from extravasation.

On the original MRI reports, 8 of the 12 cases were correctly diagnosed. Using our criteria for extravasation into an extra-articular anatomical space, 10 of the 12 cases could be diagnosed correctly. Extravasation of contrast into the QL space enabled us to detect 2 cases retrospectively that were missed on the original readings. These 2 cases also demonstrated a “J” sign. In the one case where contrast was not injected, both readers arrived at the correct diagnosis of HAGL lesion due to contrast extravasation along the humeral shaft; however there was initial discordance as to whether contrast extended into the quadrilateral space which was resolved on reanalysis. We hypothesize that had this been performed as an MR arthrogram, the presence of contrast would have been readily discerned and the initial discrepancy would have been avoided. Because there was fluid along the humeral shaft and extending off the field of view, the case was correctly diagnosed as HAGL, demonstrating the diagnostic utility of extravasation, which exploits the multiplicity of spaces through which fluid/contrast can escape the joint. In 2 cases, no extra-articular contrast was discerned in our study. Both of these cases showed the “J” sign. While the “J” sign is not diagnostic, it may be the only manifestation of such injuries and therefore should not be dismissed, particularly in the proper clinical setting.

Juxta-diaphyseal extravasation was seen only in patients with HAGL, intramuscular or paramuscular extravasation occurred only in patients with pHAGL, and QL space leakage could be seen in patients with HAGL or pHAGL lesions. While this data might suggest a propensity for leakage in certain sites for specific types of IGL injuries, we advise caution against drawing such conclusions based on this small cohort of 12 patients.

Only 5 of our 12 cases, or 42%, clearly depicted a disrupted ligament. Ideally one might expect disruption of the humeral attachment of the IGL to be diagnostic for HAGL/pHAGL on MRI; yet studies have given rise to mixed data with regard to the reliability of this finding in and of itself. This may be because investigators are using different diagnostic criteria for disruption of the ligament or because differentiation of the ligament from a portion of the capsule can be problematic. In the most recent MRI study of HAGL lesions this finding came under attack as it gave rise to false positives in both cases where it was invoked [12]. We propose a method for diagnosing HAGL lesions based on extra-articular dissemination of contrast. This analysis may be more straightforward and reproducible than attempting to differentiate a capsular rent from ligament avulsion. We further subdivided these extra-articular spaces into para/intramuscular spaces, the quadrilateral space, or the juxta-diaphyseal space with extension beyond the field of view. We excluded spaces such as the subdeltoid/subacromial bursa and subcoracoid bursa, which would have other clinical ramifications. These criteria for contrast extravasation proved to be sensitive for HAGL and one or more were observed in 83% (10 of 12) of cases.

## 6. Limitations

Limitations to this study include a relatively small cohort group of 12 patients, limiting the statistical power of our conclusions. While interobserver interpretation was studied between two musculoskeletal radiologists, a larger study could include a greater number of musculoskeletal radiologists and measure interobserver variability amongst this larger group. Other proposed ideas in a larger study could also include both musculoskeletal and nonmusculoskeletal radiologists to assess differences in interobserver variability between these two groups.

## 7. Conclusion

In conclusion, our study examined extra-articular extravasation as a method for diagnosing HAGL/pHAGL lesions. Using this analysis the positive detection rate in a cohort of arthroscopically proven HAGL/pHAGL was 83% (10 of 12). Interobserver reliability was excellent. In cases where extravasation is not present, the “J” sign, though nonspecific, may be the only evidence of subtle HAGL and pHAGL lesions.

## Figures and Tables

**Table 1:** Cohort group (all patients arthroscopically proven HAGL tears).

Age	Gender	Laterality of IGL	Anterior or posterior HAGL	Extravasation to any site		Juxtahumeral space		Quadrilateral space		Paramuscular space		Intramuscular space	
				Reader 1	Reader 2	Reader 1	Reader 2	Reader 1	Reader 2	Reader 1	Reader 2	Reader 1	Reader 2
44	Female	Right	Anterior	Yes	Yes	No	No	Yes	Yes	No	No	No	No
16	Female	Left	Anterior	No	No	No	No	No	No	No	No	No	No
32	Female	Right	Anterior and Posterior	Yes	Yes	No	No	Yes	Yes	No	No	No	No
21	Male	Left	Anterior	Yes	Yes	No	No	Yes	Yes	No	No	No	No
15	Female	Right	Anterior	Yes	Yes	Yes	Yes	Yes	Yes	No	No	No	No
19	Male	Left	Posterior^*^	Yes	Yes	No	No	No	No	Yes (IS-TM)	Yes (IS-TM)	Yes (TM)	Yes (TM)
21	Male	Left	Posterior^*^	Yes	Yes	No	No	No	No	No	No	Yes (TM)	Yes (TM)
21	Male	Right	Anterior^*^	Yes	Yes	Yes	Yes	No	No	No	No	No	No
16	Female	Right	Anterior^*^	Yes	Yes	No	No	Yes	Yes	No	No	No	No
18	Male	Right	Posterior	Yes	Yes	No	No	Yes	Yes	Yes (Ant SS)	Yes (Ant SS)	No	No
21	Male	Right	Anterior^*^	Yes	Yes	Yes	Yes	No	Yes	No	No	No	No
15	Female	Right	Posterior	No	No	No	No	No	No	No	No	No	No

TM = Teres minor.

IS-TM = Paramuscular space between infraspinatus and teres minor.

Ant SS = Paramuscular space anterior to subscapularis.

∗ = Torn ligament was directly visualized on MR arthrogram also.

Sensitivity of extravasation to any site in detecting HAGL lesions: 83%.

**Table 2:** To measure the agreement between reader 1 and reader 2 in interpreting extravasation to any site, and to a particular site, kappa statistic was used, with excellent agreement between readers amongst all sites of extravasation. Excellent agreement was defined as Kappa > 0.81 [1].

	Extravasation to any site	Juxtahumeral space	Quadrilateral space	Paramuscular space	Intramuscular space
Kappa (reader 1, reader 2)	1	1	0.833	1	1

**Table 3:** Control group (all patients arthroscopically negative for HAGL tear).

Age	Gender	Laterality of IGL tear	Extravasation to any site	
			Reader 1	Reader 2
20	Female	Right	No	No
35	Female	Right	No	No
20	Female	Right	No	No
39	Male	Right	No	No
41	Male	Right	No	No
57	Female	Right	No	No
63	Female	Right	No	No
79	Female	Left	No	No
46	Male	Left	No	No
42	Male	Left	No	No
55	Female	Left	No	No
44	Male	Left	No	No
18	Male	Left	No	No
47	Female	Left	No	No
19	Female	Right	No	No
41	Male	Right	No	No
18	Male	Left	No	No
17	Male	Left	No	No
46	Female	Left	No	No
20	Male	Right	No	No
36	Male	Right	No	No
42	Male	Right	No	No
18	Male	Right	No	No
